# “Feasibility and utility of a simple computerized test for measuring saccade latency in progressive supranuclear palsy- a proof-of-concept study”

**DOI:** 10.1186/s40734-019-0081-2

**Published:** 2019-12-06

**Authors:** Marian L. Dale, Emmi P. Scott, Saher Khalid, Andrew S. Eiseman, Travis H. Turner

**Affiliations:** 1 The Murray Center for Research on Parkinson’s Disease and Related Disorders and CurePSP Center of Care, Medical University of South Carolina, 208B Rutledge Ave MSC 108, Charleston, SC 29425 USA; 2 Department of Internal Medicine, Cleveland Clinic Akron General, Akron, OH USA; 3 Department of Ophthalmology, Medical University of South Carolina, Charleston, SC USA; 4 Oregon Health and Science University, Department of Neurology, Portland, OR USA

**Keywords:** Progressive supranuclear palsy, Saccades, Eye tracking

## Abstract

**Background:**

Reliable detection of slowed vertical saccades may help discriminate progressive supranuclear palsy (PSP) from the subset of Parkinson’s disease patients who lack tremor (akinetic-rigid or PD-postural instability and gait disorder PIGD subtype), and from age-related oculomotor changes. We investigated the feasibility of a camera-less computerized behavioral saccade latency paradigm previously validated in PD to discriminate probable PSP-Richardson syndrome (PSP-RS) from PD-PIGD and age-matched controls.

**Methods:**

In this proof-of-concept case-control study, reflexive saccade latencies were measured in 5 subjects with probable PSP-RS, 5 subjects with PD-PIGD subtype, and 5 age-matched controls using the behavioral paradigm. The battery was repeated approximately one month later. All subjects were examined off levodopa by a movement disorders neurologist and by an ophthalmologist, who also performed a dilated eye exam.

**Results:**

Vertical prosaccade latencies were longer in the PSP group (median = 903 ms) relative to PD (median = 268 ms) and control groups (median = 235 ms), with no overlap between groups (100% accuracy). PSP subjects also had larger vertical-horizontal discrepancies than comparison groups. Test-retest reliability for the behavioral saccade measures was good (interclass correlation coefficient = 0.948; 95% confidence interval [0.856, 0.982]), and the measures strongly correlated with clinical ratings.

**Conclusions:**

Computerized behavioral measurement of reflexive saccade latency is feasible in PSP, and potentially discriminates probable PSP-RS from the PD-PIGD subtype. Findings from this proof-of-concept study support utility of the approach for obtaining objective saccade metrics in clinical evaluations and for tracking change in future, larger trials of moderately advanced PSP. Future studies should also examine the behavioral paradigm in earlier presentations of PSP and other subtypes of PSP.

## Background

Supranuclear vertical eye movement paresis and palsy, eponymous features of PSP attributed to dysfunction along the medial longitudinal fasciculus of the rostral midbrain, are central in its diagnosis but can be difficult to quantify at the bedside [1]. Many of the newly-classified subtypes of possible PSP by the Movement Disorder Society 2017 criteria (and all of the probable PSP classifications) require a component of vertical supranuclear gaze palsy (“01”) or vertical saccade slowing (“O2”) [2]. Differentiating idiopathic Parkinson’s disease (PD) and PSP can be especially difficult when tremor is lacking (as in the PD-PIGD subtype) and when the axial features of PSP are less pronounced. It is also difficult to quantify changes in vertical saccades over time as PSP progresses. A logistically-simple and reliable means of quantitative saccade measurement could prove particularly beneficial for early diagnosis or as a marker of progression in PSP.

Other groups have implemented video-based eye tracking to distinguish PSP from other atypical parkinsonisms with mixed results. Using video-oculography with Eyelink (Ontario, CA), Gorges showed reduced peak eye velocities in PSP (mixed PSP-RS and PSP-parkinsonism) compared to Parkinson’s disease, MSA and healthy controls [3]. The Parkinson’s subjects had mixed akinetic-rigid and tremor-predominant phenotypes and were tested *on* dopaminergic medication. In contrast, Linder found that video-oculography could not discriminate early PSP from early PD when subjects were tested *off* dopaminergic medication [4]. Further, in moderately-advanced PSP oculomotor abnormalities beyond vertical palsy and paresis can impact feasibility of video-oculography. For example, blepharospasm, apraxia of eyelid opening, saccadic intrusions, and square wave jerks can encumber calibration and invalidate trials.

Our lab validated a computerized behavioral method to measure saccade latency without the use of video-based eye-tracking [5, 6]. The fundamental principle of the behavioral tasks is that foveal acuity is required to make fine visual perceptual judgements of a stimulus. Subjects focus on a central fixation point until a small visual target is placed in the periphery. If a saccade cannot place the fovea on the target prior to removal, perceptual discrimination cannot be made. The minimum presentation time for accurate identification (discrimination threshold) reflects the latency of the saccade (Additional file 1: Figure S1, Additional file 2: Video S1). An adaptive staircase design is used to determine the minimum presentation time of a target when reliable visual perceptions can be made (i.e., orientation of a Landolt C-optotype), and this discrimination threshold serves as proxy for saccade latency. Variations of this method have been validated in healthy adults [5], Huntington’s disease [7], and Parkinson’s disease [6], but not in PSP. In Huntington’s disease, behavioral saccade measures discriminated manifest patients from presymptomatic gene carriers, and presymptomatic gene carriers from non-gene carrier siblings [7]. In Parkinson’s disease, behavioral measures of saccade latency evinced validity with respect to measures obtained from video-oculography, demonstrated good test-retest reliability, and identified mild cognitive impairment in PD [6].

The purpose of the present study was to determine whether reflexive vertical saccade latency measured by the behavioral method could feasibly and reliably distinguish subjects with PSP-Richardson syndrome (PSP-RS) from subjects with akinetic-rigid Parkinson’s disease (PD-PIGD) and age-matched healthy controls, and to assess agreement between behavioral measures and existing clinical rating measures. In this proof-of-concept study, the goal was to first confirm that the behavioral method of reflexive saccade latency measurement with is 1) feasible in patients with PSP-RS and 2) separates well-characterized clinical groups, prior to expanding the assessment in less advanced cases of PSP or other subtypes of PSP.

## Methods

In this small, cross-sectional study we compared behavioral measures of vertical and horizontal reflexive saccades in PSP-RS, PD-PIGD and age-matched controls. We focused on the PIGD presentation of PD in order to examine populations that can be more difficult to separate clinically, since PD patients with significant resting tremor are not commonly confused with patients with PSP. We matched the groups by degree of motor impairment using the PIGD subscale of the MDS-UPDRS. Subjects underwent neurological and ophthalmologic examinations (including a dilated eye exam to rule out subclinical ocular pathology), clinical oculomotor rating measures, video-based eye tracking, and behavioral tests of vertical and horizontal reflexive saccades. Behavioral tests were repeated at a follow-up visit 1 month later to establish test-retest reliability. This study was approved by the Medical University of South Carolina Institutional Review Board (IRB# 65862/Pro00065862).

### Participants

We recruited five subjects with subjects with probable PSP-RS using the MDS-PSP criteria [2] and five subjects with Parkinson’s disease-PIGD subtype (PD-PIGD) from our university movement disorder clinic, and five age-matched healthy control subjects (HC) from the community. Diagnosis of probable PSP-RS or PD was confirmed by a movement disorder specialist (MLD). Clinical characteristics of our recruited subjects with PSP are as follows: early backward falls: 4/5 subjects, backward postural instability on exam: 5/5 subjects, apraxia of eyelid opening: 4/5 subjects, light sensitivity: 4/5 subjects, lid retraction stare: 5/5 subjects, and axial rigidity: 5/5 subjects. Radiological findings in our recruited PSP subjects included mild microangiopathy and age-appropriate volume loss in 3/5 subjects, moderate microangiopathy in the remaining 2/5 subjects, and a diminished midbrain:pons ratio in 2/5 subjects. All patients were tested *off* dopaminergic medication to compare populations without the confounder of dopaminergic medication effects. *Exclusion criteria for all subjects:* Patients with a history of deep brain simulation, traumatic brain injury, glaucoma, macular degeneration, known visual field abnormalities, oculomotor or optic nerve injury, midbrain strokes, treatment with acetylcholine augmenting or depleting pharmaceutical (such as donepezil or oxybutynin) within 60 days of testing, and use of an OTC anticholinergic (such as diphenhydramine) within 3 days of testing.

### Neurological examination

All subjects underwent cognitive screening (MoCA) [8] and eye movement ratings (completed separately by the neurologist and ophthalmologist). The clinical ratings focused on oculomotor and other ophthalmological impairments common in PSP. Items tested include square wave jerks, blink rate, lid retraction stare, gaze excursion, pursuits, saccadic velocity, apraxia of eyelid opening and closure, optokinetic response, and vestibular ocular reflex. We also performed the PSP Rating Scale (PSPRS) [9] in subjects with PSP, and the postural instability gait disturbance (PIGD) subscale of the Unified Parkinson’s Disease Rating Scale (MDS-UPDRS) [10] in all PSP and PD subjects.

### Ophthalmological examination

An experienced ophthalmologist (AE) independently completed the clinical eye ratings and performed dilated eye examinations on all subjects. Initial examination included acuity, tonometry, pupil reactivity, visual fields, extraocular movements, and refraction. Slit lamp examination inspected the anterior chamber, including the conjunctiva, cornea, iris and lens. Subjects’ eyes were dilated with 2.5% phenylephrine and 1% tropicamide for examination of the fundus including the disc, macula, and vessels.

### Behavioral saccade battery

The reflexive behavioral paradigm was administered on a PC computer with a 17″ screen using E-Prime v1.2 software (Sharpsburg, PA) [11]. Participants were seated 57 cm from the screen and instructed to keep their heads still while performing the tasks. As illustrated in Additional file 1: Fig. S1, a central fixation point was removed and a target was presented 10 degrees of visual angle above or below (vertical task) or to the left or right (horizontal task). A masking stimulus was displayed for 25 ms to reduce retinal afterimages. Participants were asked whether the Landolt C-Optotype faced forward or flipped. Responses were entered by the administrator to eliminate non-ocular motoric confounds. The tests used an adaptive staircase paradigm with 12 reversals [12]. Average presentation time for the last 10 trials was obtained as the primary outcome measure of saccade latency. This reflexive method of saccade latency measurement is less sensitive to cognitive abnormalities compared to other methods of saccade latency measurement such as volitional testing (which is similar but requires the subject to follow a cueing arrow).

### Statistical analysis

Nonparametric statistical tests (Mann-Whitney U and Wilcoxon signed rank tests) evaluated group differences on demographic and clinical measures. Completion rates were examined for behavioral tests and video-based eye tracking to assess feasibility. Video-oculography results were not included in statistical analyses due to feasibility and calibration issues in PSP subjects (described below). Correspondence between behavioral saccade measures and the neurologist’s ratings of saccade velocity in the vertical (average of upward and downward directions) and horizontal directions were examined using Pearson product-moment correlations. We chose not to combine neurologist’s and ophthalmologist’s ratings due to relatively low agreement in PSP and PD subjects (weighted Kappas = 0.531–0.667). See Additional file 1: Table S1 for inter-rater agreement for clinical ratings of saccadic velocity, gaze excursion, and eyelid opening apraxia. Test-retest reliability of the behavioral tests was evaluated with intraclass correlation coefficients (ICC) using a single-measure model for absolute agreement. SPSS version 25 (Armonk, NY: IBM Corp.) and GraphPad Prism (version 8.0, GraphPad Software Inc., La Jolla, USA) were used for statistical analysis.

## Results

### Demographic and clinical characteristics of participants

Table 1 shows the demographic and clinical characteristics of individual participants in each group. The groups were well-matched with regard to age, gender, and MDS-UPDRS PIGD score. PSP subjects had significantly lower MoCA scores than PD (*p* = 0.032) and HC subjects (*p* = 0.008). Corrected visual acuity in all subjects was at least 20/40 (Snellen-linear). The groups were well-matched with regard to basic ocular and fundoscopic examination, with the exception of punctate epithelial corneal erosions (consistent with dry eye) in three PSP and three PD subjects. See Additional file 1: Table S2 for further details on ophthalmological findings.

**Table 1:** Demographic, clinical, and oculomotor characteristics of the sample

Group	Age/Sex	MoCA	Light sensitivity	Onset (yrs)	LEDD	UPDRS PIGD	PSPRS (OM)	Saccadic velocity			Gaze excursion			Eyelid opening apraxia
								Down	Up	Hor.	Down	Up	Hor.	
PSP1	70/f	22	+++	2.5		8	43 (9)	↓↓↓	↓↓↓	↓↓↓	↓↓	↓↓↓	–	+
PSP2	67/m	20	+	3		4	28 (10)	↓↓↓	↓↓↓	↓	↓↓↓↓	↓↓↓	–	+
PSP3	68/m	24	–	4		11	43 (3)	–	↓	–	–	↓	–	+
PSP4	73/m	13	++	5		6	26 (10)	↓↓↓	↓↓↓	↓↓	↓↓↓	↓↓↓↓	–	+
PSP5	73/m	23	++	3.8		4	16 (5)	↓↓	↓↓	↓	–	↓↓↓	–	–
PD1	73/m	23	+	2.5	350	3		–	↓	–	–	↓	–	–
PD2	64/m	27	–	12	1065	8		–	↓	–	–	–	–	–
PD3	64/f	29	+	2.5	550	3		↓	–	–	–	–	–	–
PD4	63/f	25	–	4	350	1		–	–	–	–	–	–	–
PD5	72/f	24	–	2.5	300	1		–	↓	–	–	–	–	–
HC1	69/m	27	+					–	↓	–	–	–	–	–
HC2	72/f	29	+					–	↓	–	–	–		
HC3	74/f	26	+					–	–	–	–	–	–	–
HC4	71/m	27	–					–	–	–	–	–	–	–
HC5	74/f	29	–					–	–	–	–	–	–	–

Abbreviations: *PSP* Progressive supranuclear palsy, *PD* Parkinson’s disease, *HC* Healthy controls, *MoCA* Montreal Cognitive Assessment, *(LEDD)* Levodopa equivalent daily dose, *UPDRS PIGD* Unified Parkinson’s Disease Rating Scale- Postural instability/gait difficulty subscale, *PSPRS (OM)* PSP Rating Scale- Oculomotor subscale, Hor. = horizontal.Light sensitivity reported by participants as - = none, + = mild, ++ = moderate, +++ = severe. Saccadic velocity (neurologist ratings): - = normal, ↓ = mildly slowed, ↓↓ = moderately slowed, ↓↓↓ = severely slowed. Gaze excursion (neurologist ratings): - = no limitation, ↓ = 86–100% of normal excursion, ↓↓ = 51–85% of normal excursion, ↓↓↓ = 16–50% of normal excursion, ↓↓↓↓ = < 15% of normal excursion. Apraxia (neurologist ratings): + = present, − = absent

### Feasibility

All subjects were able to complete behavioral tests of vertical and horizontal prosaccades at both time points. Two PSP subjects and one PD subject required bifocals but they were not outliers with respect to vertical-horizonal differences or group differences in vertical saccade latency. For consistency these participants used bifocals for both behavioral testing visits.

**Fig. 1:**
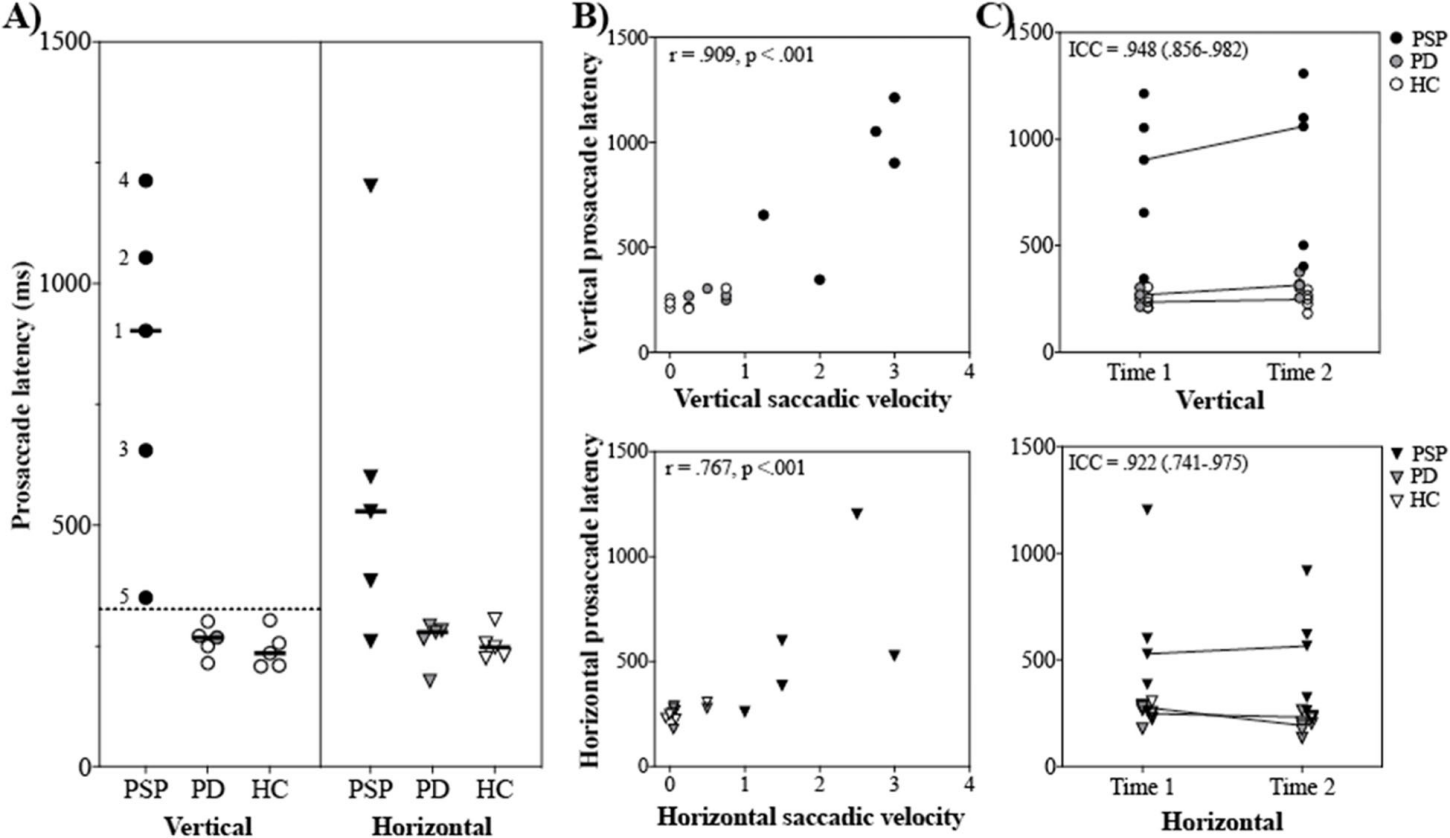
**a** Performance on behavioral tests of vertical (left) and horizontal (right) prosaccade latency. Solid horizontal lines represent group medians. For the vertical prosaccade tests, PSP subject numbers are provided, and the dotted line represents the cut-off (between 305 and 345 ms) that separates PSP subjects from PD and HC subjects with 100% accuracy. **b** Concordance between behavioral tests and clinical ratings of vertical (top) and horizontal (bottom) saccades. **c** Test-retest reliability and stability of performance on vertical (top) and horizontal (bottom) prosaccade tests. Dots represent individual data points and lines connect group medians

Although all PD and HC subjects were able to complete comparative video-based eye tracking, apraxia of eyelid opening and inability to steadily fixate on targets resulted in failed calibration or invalid data for the eye tracking tests in 3/5 PSP subjects (subjects 1, 2, and 4, who had the most severe oculomotor impairments according to Table 1 and Fig. 1a). Performance was considered invalid if over half of eye tracking trials were unreadable.

f

### Latency of prosaccades in PSP, PD and controls

Vertical prosaccade latencies were longer in the PSP group (median = 903 ms) relative to PD (median = 268 ms) and HC groups (median = 235 ms). Figure 1a shows that there were no overlapping data points between groups on the vertical prosaccade test, indicating that a projected cut-off score between 305 and 345 ms would accurately discriminate 100% of PSP and PD subjects. As shown in Fig. 1a, differences in horizontal saccades between PSP (median = 528.5 ms) and PD (median = 280.5 ms) were not readily apparent. Vertical prosaccade latencies were substantially longer than horizontal prosaccade latencies in four out of five PSP subjects (median difference = 374 ms, 95% CI [40.0, 453.0]). These discrepancies were not observed in PD (median difference = 9.0 ms, 95% CI [21.0, 37.0]) or HC subjects (median difference = 12.5 ms, 95% CI [− 21.0, 1.0]).

### Correlation with clinical variables

As shown in Fig. 1b, behavioral tests of prosaccade latency showed good concordance with clinical ratings of saccadic velocity in both vertical (r = 0.909, *p* < 0.001) and horizontal directions (r = 0.767, *p* < 0.001) in the total sample. Correlations with clinical ratings of vertical saccades remained significant after controlling for MoCA scores (r = 0.642, *p* = 0.013), suggesting minimal influence of cognitive factors on behavioral task performance.

### Test-retest reliability of behavioral saccade measures in PSP

Both vertical and horizontal behavioral tests demonstrated good test-retest reliability in the total sample, which is shown in Fig. 1c (vertical ICC = 0.948; 95% CI [0.856, 0.982]; horizontal ICC = 0.922, 95% CI [0.741, 0.975]). Figure 1c Also illustrates stability of individual performance across both time points without indication of practice effects.

## Discussion

In this cross-sectional study of 15 subjects, we found that reflexive vertical prosaccade latency detected by the computerized behavioral paradigm discriminated probable PSP-RS from Parkinson’s disease (PIGD subtype) and healthy age-matched controls. This pilot study is the first to discriminate subjects with PSP from the PD-PIGD subtype using behavioral saccade measures. We tested all subjects off levodopa, allowing for a more equal comparison between groups, and included a dilated fundus examination to rule out confounding subclinical ocular pathologies. We also found that behavioral measures correlate strongly with existing clinical scales for eye movement abnormalities in PSP and demonstrate good test-retest reliability.

The ability of computerized behavioral measurements of vertical reflexive saccade latency to reliably discriminate probable PSP-RS from PD-PIGD subtype in this small, proof-of-concept study has practical importance. Clinical ratings of saccades are subject to variation between raters at milder levels of impairment (Additional file 1: Table S1). Unlike ordinal scales, the computerized behavioral paradigm mimics saccade testing in the clinic, but provides a continuous quantitative measure that could prove useful in clinical trials as a marker of disease progression. Behavioral testing of reflexive vertical saccade latency can be implemented in less than 5 min and requires minimal training and a standard PC or laptop computer.

The present study is small, cross-sectional, and lacks histological confirmation of the diagnoses. We included only probable PSP-Richardson syndrome for this for proof-of-concept investigation. Future studies are needed to examine behavioral saccade measures in PSP patients earlier in their disease course. The fact that vertical prosaccade scores did not overlap at all between moderately advanced PSP and PD patients in our study is promising; however, future studies with larger samples of patients at earlier disease stages are needed to determine whether these tests could aid in differential diagnosis. With larger studies a reliable cut-point could be derived to facilitate early differential diagnosis, particularly in possible-PSP subtypes without obvious saccadic abnormalities on neurological exam. The variability in vertical prosaccades across our PSP subjects indicates that the behavioral measures are sensitive to a wide range of impairment; however, longitudinal studies are needed assess association of behavioral saccade measures and PSP progression. A 5-year longitudinal study of 30 patients with PSP is currently under development to determine the extent to which the behavioral saccade measures reliably track disease progression across one-year intervals.

Despite the limitations of this small pilot study, we found that the behavioral prosaccade tests 1) were feasible and relatively robust to cognitive and oculomotor dysfunction in PSP, 2) differentiated probable PSP-RS patients from PD and normal controls, 3) correlated strongly with clinical outcomes associated with disease progression, and 4) produced reliable and stable scores from baseline to 1-month follow-up. There is a pressing need to identify biomarkers/surrogate outcome measures for PSP clinical trials that correlate with clinical measures of disease progression and can be administered longitudinally. Our findings suggest strong test-retest reliability (and no ceiling/floor effects) and correlation with clinical measures, but larger longitudinal studies could better determine the value of these measures in tracking disease progression.

## Conclusions

Findings from this preliminary study support utility of the computerized behavioral saccade paradigm for easily obtaining objective saccade metrics in clinical evaluations and for tracking change in future trials of moderately advanced PSP.

## Supplementary information

**Additional file 1: Figure S1.** Example screenshots for the vertical prosaccade (top) and horizontal prosaccade tasks are illustrated. **Table S1.** Inter-rater agreement for clinical ratings in PSP and PD subjects (*n* = 10). **Table S2.** Frequency of abnormal ophthalmologic findings in each group.

**Additional file 2:.** **Video S1.** Example video of a patient with PSP performing the refexive vertical saccade behavioral testing: The patient is asked to note whether the presented "C" stimulus is in the regular, forward orientation ("same") or is facing backward ("flipped"). The examiner records responses on the keyboard to eliminate confounding hand coordination or motor reaction deficits.

## Data Availability

The datasets during and/or analyzed during the current study available from the corresponding author on reasonable request.

## Abbreviations

HC: Healthy controls
ICC: Intraclass correlation coefficients
MDS-UPDRS: Movement Disorders Society Unified Parkinson’s Disease Rating Scale
MoCA: Montreal Cognitive Assessment
MSA: Multiple system atrophy
OTC: Over the counter
PD: Parkinson’s disease
PD-PIGD: Parkinson’s disease- postural instability and gait difficulty
PSP: Progressive supranuclear palsy
PSPRS: Progressive supranuclear palsy rating scale
PSP-RS: Progressive supranuclear palsy- Richardson’s syndrome
