# Supplementary material for: “Feasibility and utility of a simple computerized test for measuring saccade latency in progressive supranuclear palsy- a proof-of-concept study”
Source: J Clin Mov Disord. 2019 Dec 6;6:6. doi: 10.1186/s40734-019-0081-2 (PMC6896714; doi:10.1186/s40734-019-0081-2)
Supplement: Supplementary file 1 — Additional file 1: Figure S1. Example screenshots for the vertical prosaccade (top) and horizontal prosaccade tasks are illustrated. Table S1. Inter-rater agreement for clinical ratings in PSP and PD subjects (n = 10). Table S2. Frequency of abnormal ophthalmologic findings in each group. [file 40734_2019_81_MOESM1_ESM.docx]

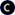

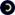

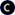

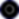

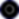

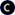


Was the target facing the same direction or did it flip?

Target Presentation

Mask (25ms)

Response

Fixation

Was the target facing the same direction or did it flip?

Supplementary Figure 1. Example screenshots for the vertical prosaccade (top) and horizontal prosaccade tasks are illustrated. Participants are seated 57cm from the screen. A central fixation point with a Landold-C optotype subtending 1 degree of visual angle is presented for 1000ms. In the Vertical prosaccade task, the fixation point is removed at the same moment a target is presented either 10cm above or below; in the horizontal task the target is presented 10cm to the left or right. Foveal acuity is required to make perceptual discrimination as to whether the target has remained facing the same direction or flipped. If a prosaccade cannot place the fovea onto the target before it is removed, the participant will be unable to reliably state whether it has flipped. Target presentation time is progressively changed following an adaptive staircase design until a discrimination threshold is reached at 12 reversals. In this way, discrimination threshold reflects saccade latency.

| Supplementary Table 1. Inter-rater agreement for clinical ratings in PSP and PD subjects (n = 10) | | | | | | | |
| --- | --- | --- | --- | --- | --- | --- | --- |
| **Saccadic Velocity** | | | | | | | |
|  | |  | Ophthalmologist rating | | | | Total |
| Downward | | Neurologist rating | Normal | Mildly slowed | Moderately slowed | Severely slowed |  |
| Weighted κ = 0.577  (0.270-0.885) | | Normal | **1** | 3 | 1 | 0 | 5 |
|  |  | Mildly slowed | 1 | **0** | 0 | 0 | 1 |
|  |  | Moderately slowed | 0 | 0 | **1** | 0 | 1 |
|  |  | Severely slowed | 0 | 0 | 0 | **3** | 3 |
|  | Total | | 2 | 3 | 2 | 3 | 10 |
|  | | | | | | | |
|  |  | | Ophthalmologist rating | | | | Total |
| Upward | Neurologist rating | | Normal | Mildly slowed | Moderately slowed | Severely slowed |  |
| Weighted κ = 0.667  (0.395-0.939) | | Normal | **1** | 1 | 0 | 0 | 2 |
|  |  | Mildly slowed | 1 | **2** | 1 | 0 | 4 |
|  |  | Moderately slowed | 0 | 0 | **1** | 0 | 1 |
|  |  | Severely slowed | 0 | 0 | 1 | **2** | 3 |
|  | Total | | 2 | 3 | 3 | 2 | 10 |
|  | | | | | | | |
|  |  | | Ophalmologist rating | | | | Total |
| Horizontal | Neurologist rating | | Normal | Mildly slowed | Moderately slowed | Severely slowed |  |
| Weighted κ = 0.531  (0.212-0.851) | | Normal | **4** | 1 | 1 | 0 | 6 |
|  |  | Mildly slowed | 0 | **0** | 2 | 0 | 2 |
|  |  | Moderately slowed | 0 | 0 | **0** | 1 | 1 |
|  |  | Severely slowed | 0 | 0 | 0 | **1** | 1 |
|  | Total | | 4 | 1 | 3 | 2 | 10 |

| **Gaze Excursion** |  | Ophthalmologist rating | | | | | |  |
| --- | --- | --- | --- | --- | --- | --- | --- | --- |
| Downward | Neurologist rating | No limitation | 86-100% | 51-85% | 16-50% | <15% | Total | |
| Weighted *κ* = 0.647  (0.319-0.976) | No limitation | **6** | 0 | 0 | 1 | 0 | 7 | |
|  | 86-100% | 0 | **0** | 0 | 0 | 0 | 0 | |
|  | 51-85% | 0 | 0 | **0** | 0 | 1 | 1 | |
|  | 16-50% | 0 | 0 | 0 | **0** | 1 | 1 | |
|  | <15% | 0 | 0 | 0 | 0 | **1** | 1 | |
|  | Total | 6 | 0 | 0 | 1 | 3 | 10 | |
|  |  |  |  |  |  |  |  | |
|  |  | Ophthalmologist rating | | | | |  | |
| Upward | Neurologist rating | No limitation | 86-100% | 51-85% | 16-50% | <15% | Total | |
| Weighted *κ* = 0.828  (0.600-1.000) | No limitation | **4** | 0 | 0 | 0 | 0 | 4 | |
|  | 86-100% | 0 | **1** | 0 | 1 | 0 | 2 | |
|  | 51-85% | 0 | 0 | **0** | 0 | 0 | 0 | |
|  | 16-50% | 0 | 0 | 0 | **2** | 1 | 3 | |
|  | <15% | 0 | 0 | 0 | 0 | **1** | 1 | |
|  | Total | 4 | 1 | 0 | 3 | 2 | 10 | |
|  |  |  |  |  |  |  |  | |
|  |  | Ophthalmologist rating | | | | |  | |
| Horizontal | Neurologist rating | No limitation | 86-100% | 51-85% | 16-50% | <15% | Total | |
| Weighted *κ* = N/A  due to restricted  range | No limitation | **6** | 2 | 1 | 1 | 0 | 10 | |
|  | 86-100% | 0 | **0** | 0 | 0 | 0 | 0 | |
|  | 51-85% | 0 | 0 | **0** | 0 | 0 | 0 | |
|  | 16-50% | 0 | 0 | 0 | **0** | 0 | 0 | |
|  | <15% | 0 | 0 | 0 | 0 | **0** | 0 | |
|  | Total | 6 | 2 | 1 | 1 | 0 | 10 | |

| **Apraxia of eyelid opening** | | | | |
| --- | --- | --- | --- | --- |
|  | | Ophthalmologist rating | | Total |
|  | Neurologist rating | Absent | Present |  |
| *κ* = 0.348 (0.000-0.937) | Absent | **5** | 1 | 6 |
|  | Present | 2 | **2** | 4 |
|  | Total | 7 | 3 | 10 |

| Supplementary Table 2. Frequency of abnormal ophthalmologic findings in each group | | | |
| --- | --- | --- | --- |
| Findings | PSP | PD | HC |
| Ophthalmologic exam |  |  |  |
| Tonometry | 1^1^ | 0 | 0 |
| Cornea^2^ | 3 | 3 | 2 |
| Lens^3^ | 2 IOL; 3 2+NS | 4 2+NS | 2 IOL; 3 2+NS |
| Disc | 0 | 0 | 0 |
| Macula^4^ | 0 | 1 | 0 |

PSP = progressive supranuclear palsy; PD = Parkinson’s disease; HC = healthy controls.

^1^Pressure of > 30 mmHg consistent with untreated glaucoma.

^2^Abnormal cornea findings for PSP, PD, and 1 HC subject were inferior punctate epithelial erosions. 1 HC subject had superior epithelial irregularity with mild stromal haze.

^3^ NS = nuclear sclerosing cataracts (consistent with aging); IOL = intraocular lens (cataract surgery)/posterior chamber intraocular lens

^4^ RPE = retinal pigment epithelium mottling
